# *Cocculus hirsutus* (L.) W.Theob. (Menispermaceae): A Review on Traditional Uses, Phytochemistry and Pharmacological Activities

**DOI:** 10.3390/medicines7110069

**Published:** 2020-11-10

**Authors:** Rajan Logesh, Niranjan Das, Anjana Adhikari-Devkota, Hari Prasad Devkota

**Affiliations:** 1 TIFAC CORE in Herbal Drugs, Department of Pharmacognosy and Phytopharmacy, JSS College of Pharmacy, JSS Academy of Higher Education & Research, Rockland’s, Ooty 643001, Tamil Nadu, India; rlogesh14@gmail.com; 2 Department of Chemistry, Iswar Chandra Vidyasagar College, Belonia 799155, Tripura, India; ndnsmu@gmail.com; 3 Graduate School of Pharmaceutical Sciences, Kumamoto University, 5-1 Oe-honmachi, Chuo-ku, Kumamoto 862-0973, Japan; anjana@kumamoto-u.ac.jp; 4 Program for Leading Graduate Schools, Health life Sciences: Interdisciplinary and Glocal Oriented (HIGO) Program, 5-1 Oe-honmachi, Chuo-ku, Kumamoto 862-0973, Japan

**Keywords:** *Cocculus hirsutus*, Menispermaceae, Jamti-ki-bel, traditional uses, alkaloids, pharmacological activity

## Abstract

**Background:***Cocculus hirsutus* (L.) W.Theob. (Menispermaceae) is a perennial climber distributed mostly in tropical and subtropical areas. The main aim of this article is to collect and analyze the scientific information related to traditional uses, bioactive chemical constituents and pharmacological activities. **Methods:** Scientific information on *C. hirsutus* was retrieved from the online bibliographic databases (e.g. MEDLINE/PubMed, SciFinder, Web of Science, Google Scholar and Scopus). Information regarding traditional uses was also acquired from secondary resources including books and proceedings. **Results:** Different plant parts of *C. hirsutus* were reported to be used for the treatment of fever, skin diseases, stomach disorders and urinary diseases. Alkaloids such as jasminitine, hirsutine, cohirsitine and their derivatives along with a few flavonoids, triterpene derivatives and volatile compounds were reported from whole plant or different plant parts. Extracts were evaluated for their antimicrobial, antidiabetic, immunomodulatory and hepatoprotective activities among others. **Conclusion:** Although widely used in traditional medicines, only a few studies have been performed related to chemical constituents. Most of the biological activity evaluations were carried out using in vitro evaluation methods and only a few studies were carried out in animal models. In the future, properly designed in vivo and clinical studies are necessary to evaluate the pharmacological activities of *C. hirsutus* along with bioassay-guided studies to isolate and identify the active constituents.

## 1. Introduction

**Figure 1:**
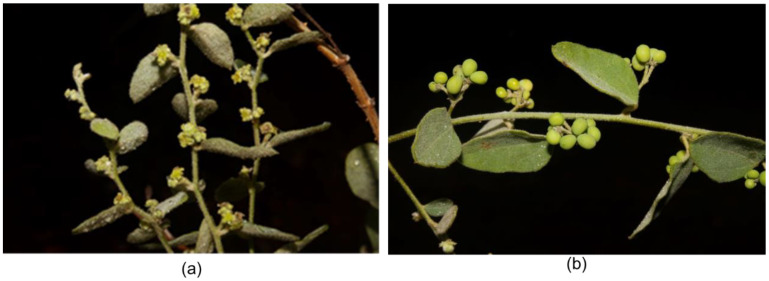
Photographs of male (**a**) and female (**b**) plants of *Cocculus hirsutus* (Photos by Dr. D. Narasimhan and Mr. K. Devanathan).

**Figure 2:**
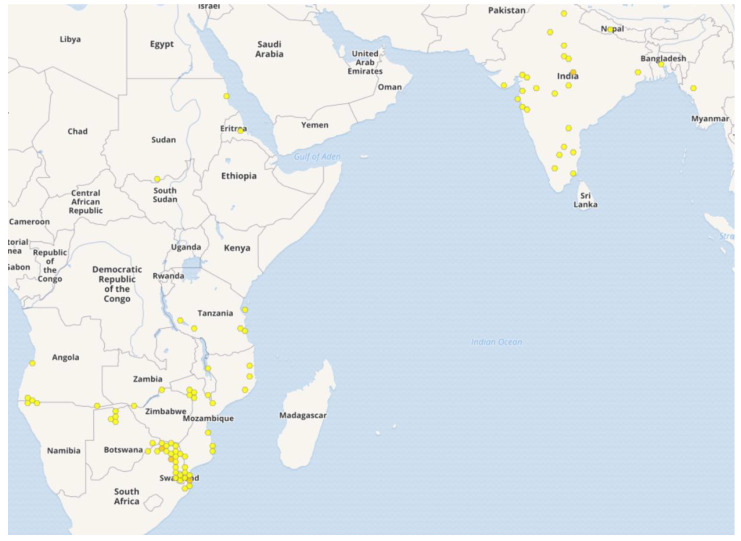
Distribution of *C. hirsutus* in Asia and Africa. (Source: GBIF, https://www.gbif.org/species/7930800 [7]).

The Menispermaceae family consists of about 70 genera and 500 species [1]. Among them, the genus *Cocculus* comprises about 10 species distributed in Asia, Africa, Australia and North America [1]. *Cocculus hirsutus* (L.) W.Theob (Figure 1) (syn. *Cebatha hirsuta* (L.) Kuntze, *Cebatha villosa* C.Chr., *Cocculus aristolochiae* DC., *Cocculus hastatus* DC., *Cocculus hirsutus* (L.) Diels, *Cocculus holopeira-torrida* Broun & R.L.Massey, *Cocculus linnaeanus* Kurz, *Cocculus sepium* Colebr., *Cocculus villosus* DC., *Menispermum hirsutum* L.) [2] is a perennial climber distributed mostly in tropical and subtropical areas [3]. In Asia, it is reported from India, Myanmar, Nepal and Pakistan and southern China [1,4,5,6]. In Africa, it is reported from Egypt, Sudan and Eritrea, Angola and south-west and southern Africa [1,6]. The detailed distribution in Asia and Africa is given in Figure 2 [7]. It is known by various names in local languages such as: Broom creeper (English); Huyer (Bengali); Farid buti, Jamti ki bel (Hindi); Kaage Mari (Kannada); Farid buti (Urdu); Paathaalagarudakkoti (Malayalam), Kaanse laharo (Nepali); Garudi, Patalagarudi (Sanskrit), Chipuru-tiga (Telegu); Kattu-k-koti (Tamil) among others [3,4,8]. 

Various plant parts of *C. hirsutus* are widely used in South Asia for the treatment of fever, rheumatism, skin disorders and visceral diseases and also as a detoxifier [9]. In Africa, stems are used to make baskets and the purple berries are eaten or used to dye basket materials. Leaves are used by Tsonga people as an important herb in their diet [1]. Regarding the pharmacological activity evaluations, extracts obtained from the plant parts of *C. hirsutus* have shown potent diuretic, laxative [10], analgesic and anti-inflammatory activities [11]. Mainly alkaloids and some other compounds are reported from whole plant or different plant parts, however the detailed chemical analysis has not been performed yet. 

A current pandemic outbreak of Coronavirus diseases-2019 (COVID-19) has affected 216 countries and territories and more than 47 million cases were reported till 3 November 2020 (https://www.worldometers.info/coronavirus/). Along with the research on the development of vaccines and antiviral drugs, many researchers are also focusing on the plant-derived natural products as potential sources of therapeutic agents. According to Clinical Trials Registry-India [12], an open label, randomized, comparative, multi-center, parallel group, controlled clinical study has been started in India to evaluate the effect and safety of aqueous extract of *Cocculus hirsutus* (AQCH) tablets in treatment of Coronavirus infection (CTRI Number: CTRI/2020/05/025397, registered on 28 May 2020). Previously, a randomized, Phase-I, double-blind, placebo-controlled, dose-escalation study to evaluate safety and tolerability of AQCH tablets in healthy adult human subjects was registered (CTRI Number: CTRI/2019/12/022297, registered on 10 December 2019). To the best of our knowledge, the outcomes of these studies have not been published yet. 

For a medicinal plant species that has been widely used in traditional medicines and is also being considered as a source for the development of therapeutic agents for various diseases, it is important to critically analyze and understand the available scientific information about the chemical constituents and pharmacological activities. Thus, the aim of this article is to compile and analyze the available scientific information about these reported aspects of *C. hirsutus.* Persistent gaps in research and future perspectives on research and utilization of *C. hirsutus* are also discussed in detail. 

## 2. Traditional Uses

Various publications have mentioned the traditional uses of *C. hirsutus* as practiced by the ethnic people in South Asia. The Koyas use the leaf paste, which is applied on head for its cooling effect [13,14,15,16,17]. The juice of the plant mixed with sesame oil is applied on the head and body to reduce heat. To allay the stomach heat and for the treatment of blood dysentery, the plant-paste is applied over the navel region [16]. The leaves are used to treat prurigo, impetigo, eczema, sores, cuts, wounds and other skin disorders [18,19]. Leaves are also used in the treatment of urine disorders, fever, leucorrhoea and acute gonorrhea [17,20,21,22]. The leaves and stems are used in the treatment of conjunctivitis and other eye disorders [15,18,23]. The leaf powder is given orally for the treatment of dysentery and diarrhea [24]. The stem is used in the treatment of stomach disorders [16,18]. 

The roots are bitter, alterative and laxative and are used in the treatment of fever, skin irritation, rheumatism, gout, syphilitic cachexia and also in children for the treatment of stomach-ache [18,21,22,25,26,27,28,29,30,31]. The extract of stems and roots are used as a sedative, hypotensive, cardiotonic and spasmolytic [25]. The root is made into paste and mixed with water and given orally to reduce stomach pain [32].

## 3. Chemical Constituents

**Figure 3:**
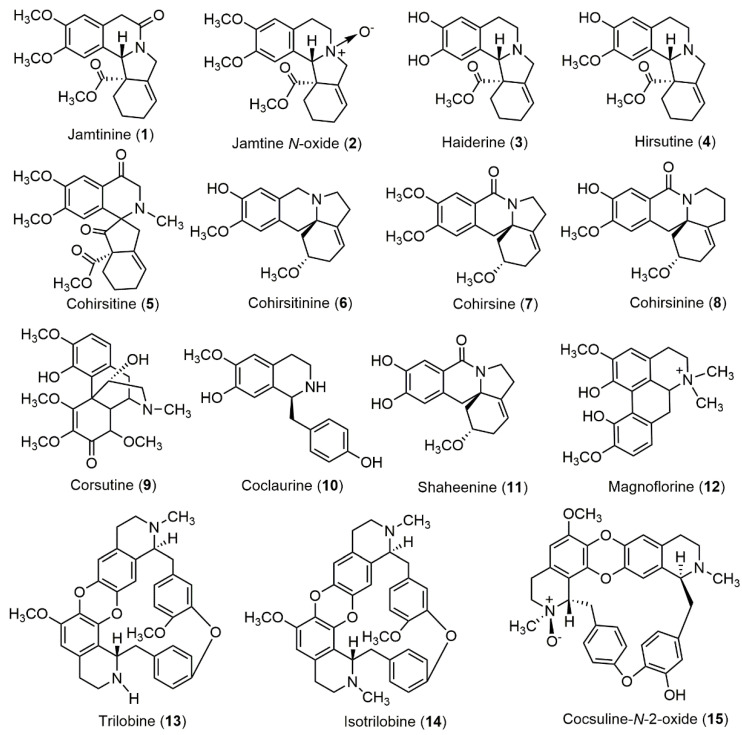
Structures of alkaloids reported from *C. hirsutus.*

Although widely used in traditional medicines and studied well for its pharmacological activities, the chemical constituents of *C. hirsutus* are not well explored. Earlier studies in 1960s and 1970s have reported the presence of alkaloids through preliminary phytochemical screenings and isolation and identification of a few alkaloids such as trilobine, isotrilobine, coclaurine and magnoflorine [33,34,35] and other compounds i.e., *β*-sitosterol, ginnol and monomethyl ether of inositol [36]. Few studies performed during or after 1980s, have reported several alkaloids from various plant parts. The list of alkaloids isolated from *C. hirsutus* is given in Table 1 and their structures are represented in Figure 3. Three flavonoids rutin, liquiritin and quercetin were also reported from the leaves [37]. A triterpene derivative, hirsudiol is also reported from the ethanol extract of whole plant [38]. Similarly, *β*-sitosterol and 28-acetyl botulin were isolated from the aqueous extract of aerial parts [39]. Many studies have reported the preliminary phytochemical screening of the extracts and presence of carbohydrates, steroids, alkaloids, glycosides, flavonoids, tannins and saponins [40]. Further, gas chromatography-mass spectroscopy (GC-MS) analysis of the extracts have revealed the presence of various compounds [40,41]. 

## 4. Pharmacological Activities

Various pharmacological activities have been reported for the extracts and isolated compounds from the different plant parts of *C. hirsutus*.

### 4.1. Anti-Microbial Activity

Jethva et al. carried out the anti-mycobacterial activity of the aqueous extract of *C. hirsutus* against *Mycobacterium tuberculosis* H_37_Rv and the extract showed potent anti-mycobacterial activity with the inhibition percentage of 80.26% [49]. Gupta et al. evaluated the anti-mycobacterial activity of ethanol extract of the leaves of *C. hirsutus* against *M. tuberculosis* H_37_Rv and various multidrug resistant (MDR) strains. The extract showed potent anti-mycobacterial activity against *M. tuberculosis* H37Rv and MDR strains JAl-19187, JAL-19126, JAL 19049, JAL 19111 and JAL- 19188 with MIC values of 500, 250, 500, 250, 500 and 500 µg/mL, respectively [50].

Devi et al. carried out the anti-bacterial activity of methanol, ethanol and aqueous extract of the leaves of *C. hirsutus* (a concentration of 25, 50, 75, 100 µL) using clinical bacterial isolates such as *Escherichia coli, Salmonella typhi, Micrococcus luteus, Staphylococcus aureus, Acetobacter laffi, Proteus mirabilis* and *Bacillus cereus* and the extracts showed potent antibacterial activities [51]. Nayak and Singhai carried out the anti-bacterial activity evaluation of the different extracts of roots of *C. hirsutus* against *Staphlococcus aureus, Escherchia coli, Pseudomonas aeruginosa* and *Salmonella typhi* and the ethanol extract showed potent antibacterial activity [52]. 

Devi et al. evaluated the anti-fungal activity of the aqueous extract of *C. hirsutus* against *Rhizopus arrhizus, Sclerotium rolfsii* and *Fusarium solani* fungal strains and the extract showed antifungal activity against *S. rolfsii* and *F. solani* [53].

### 4.2. Anti-Malarial and Insecticidal Activity

Brahmam and Sunita carried out the in-vitro antimalarial activity of different extracts of roots of *C. hirsutus* against two *Plasmodium falciparum* strains, i.e., 3D7 (chloroquine sensitive strain) and K1 (chloroquine resistance strain). The chloroform and methanol extracts showed potent activity against both strains. [54]. Elango et al. evaluated the larvicidal activity of the leaves of *C. hirsutus* against malaria vector *Anopheles subpictus* larvae and the different extracts showed potent activity with a percentage mortality at 24 h and emergence inhibition values: hexane extract (60 ± 2.04 and 75 ± 2.44), choloroform extract (78 ± 2.56 and 85 ± 1.50), ethyl acetate extract (86 ± 1.29 and 69 ± 1.71), acetone extract (100 ± 0.00 and 68 ± 2.13) and methanol extract (81 ± 1.08 and 100 ± 0.00) [55]. Elango et al. also reported the larvicidal activity of the ethyl acetate and acetone extracts of the leaves of *C. hirsutus* against *Culex tritaeniorhynchus* and *Anopheles subpictus* [56]. 

### 4.3. Anti-Cancer Activity

De Wet et al. carried out the anti-cancer activity of crude alkaloidal extract of rhizomes of *C. hirsutus* in three cancer cell lines, i.e., breast (MCF7), melanoma (UACC62) and renal (TK10) cell lines and the extract showed moderate anticancer activity [57]. Thavamani et al. carried out the in-vitro cytotoxic activity of the methanolic extract of *C. hirsutus* against HeLa cell line and the results showed an IC_50_ value of 111 µg/mL [58]. Another study evaluated the anti-cancer activity of the plant *C. hirsutus* against Dalton’s lymphoma ascites (DLA) cells in mice. The methanolic extract of *C. hirsutus* showed significant cytotoxic activity with an IC_50_ value of 84.56 mg/mL in MCF-7 cancer cell line in-vitro. The extract also showed in-vivo antitumor activity as the doses of 200 and 400 mg/kg body weight significantly reduced the packed cell volume, tumor cell count, and restored the hematological and serum biochemical parameters towards the normal values [59].

### 4.4. Immunomodulatory Activity

Mallik and Nayak evaluated the immunomodulatory activity of the combination (1:1, 2:1 and 1:2 ration) of leaves of *C. hirsutus* and flowers of *Sesbania grandiflora* (L.) Pers. (Fabaceae) in mice. The 1:1 combination mixture showed potent immunostimulatory activity [60]. Rastogi et al. evaluated the immunostimulatory activity of aqueous and ethanolic extract of aerial parts of *C. hirsutus* in normal as well as cyclophosphamide induced immunosuppressed rats. The extracts showed an dose dependent increase in the carbon clearance, humoral antibody (HA) titre, delayed type hypersensitivity (DTH) and white blood cell (WBC) count in a dose dependent manner and authors concluded that the extract was effective to stimulate the immune system and also to protect from the immunosuppressant [61].

### 4.5. Anti-Diabetic Acitivty

Badole et al. reported the anti-hyperglycemic activity evaluation of aqueous extract of leaves of *C. hirsutus* in alloxan-induced diabetic mice. The oral administration of the extract at the dose of 250, 500, and 1000 mg/kg showed significant decrease in serum glucose level at 28th day of administration. In the oral glucose tolerance test (OGTT) in normal mice, the oral administration of extract (1000 mg/kg ) increased the glucose tolerance [62]. Sangameswaran and Jayakar evaluated the anti-diabetic of *C. hirsutus* in streptozotocin-induced diabetic rats and the oral administration of methanolic extract (400 and 800 mg/kg) showed decrease in the blood glucose level [63].

### 4.6. Anti-Oxidant Activity

Srikanta and Dharmesh evaluated the anti-oxidant activity of the aqueous extract of the leaves of *C. hirsutus* using 1,1-diphenyl-2-picrylhydrazyl (DPPH) free radical and reducing power assay, and the extract showed potent DPPH free radical scavenging assay with IC_50_ values of 2.75 ± 0.3 μg gallic acid equivalents (GAE)/ml and reducing power activity with the value of 65.17 ± 4.8 U/mg GAE. The extract also showed the total phenolic content of 31.83 ± 3.1 mg GAE/g [64]. Rakkimuthu et al. studied the anti-oxidant activity of *C. hirsutus*. The results showed potent DPPH free radical scavenging activity, ABTS free radical scavenging activity, nitric oxide scavenging activity, reducing power, inhibition of lipid peroxidation and metal chelating activity assay as ascorbic acid [65].

### 4.7. Hepatoprotective Activity

Thakare et al. evaluated the hepatoprotective activity of the methanolic extract of *C. hirsutus* in albino Wister rats with ethanol-induced hepatotoxicity and the oral administration of the extract at doses of 100, 200 and 400 mg/kg significantly lowered the levels of AST, ALT, ALP, LDH, direct and total bilirubin and cholesterol [66].

### 4.8. Diruetic and Nephroprotective Activity

Ganapaty et al. studied the diuretic activity of the aqueous extract of the aerial parts of *C. hirsutus* in normap mice and the extract at a dose of 100 and 200 mg/kg, p.o. showed significant increase in the urinary concentrations of Na^+^ and K^+^ suggesting potent diuretic activity [67]. Badole et al. carried out the acute and chronic diuretic activity of the ethanolic extract of leaves of *C. hirsutus* in normal rats and the extract significantly increased the urinary concentrations of Na^+^ and K^+^ [68]. Gadapuram et al. reported the potent nephroprotective activity of the ethanolic extract of the leaves of *C. hirsutus* in 5/6 nephrectomized rat model [69].

### 4.9. Other Activities

Ranjan et al. evaluated the wound healing activity from the leaves of *C. hirsutus* and reported that the methanolic extract showed the highest wound healing activity among the tested groups when compared to the standard [70]. Ganapaty et al. evaluated the laxative activity of the aqueous extract of the aerial parts and the extract showed significant laxative activity at the doses of 100 and 200 mg/kg, p.o. [67]. Sangameswaran and Jayakar evaluated the spermatogenic activity *C. hirsutus* in streptozotocin-induced diabetic rats and the increase in the sperm count was observed at a concentration of 400 mg/kg p.o (102.83 ± 1.85) and 800 mg/kg p.o. (117.83 ± 3.49) when compared to the normal group (74.83 ± 1.97) [63]. Elango and Rahuman reported the potent anti-parasitic activity of extracts of leaves of *C. hirsutus* against veterinary ticks and fluke [71].

## 5. Toxicological Studies 

Ganapaty et al. evaluated the acute toxicity of the aqueous extract of the aerial parts of *C. hirsutus* in mice after oral administration of the extract at several dose ranges from of 100 to 3000 mg/kg. The extract showed sedative effect and increased urination and defecation at all doses, however no mortality was observed until 14 days after administration [67]. 

## 6. Patents 

Some patents were also registered for the use of *C. hirsutus* formulations for their potential use in treatment of various diseases. For example, the patents related to the use of extract of *C. hirsutus* in prevention and treatment of dengue, the components of extract and the formulation were registered [72,73]. Similarly, patents related to the formulation and use of *C. hirsutus* alone or in combination with other medicinal plants for the treatment of tuberculosis [74] and respiratory tract diseases [75] are also registered.

## 7. Conclusions

*Cocculus hirsutus* is widely used in various traditional medicine systems in South Asia for the treatment of fever, skin diseases, stomach disorders, urinary diseases and also as a sedative among many other uses. The most commonly reported constituents were alkaloids such as jasminitine, hirsutine, cohirsitine and their derivatives. Some flavonoids, triterpene derivatives and volatile compounds were also reported from different plant parts. However, these chemical isolation studies were mostly carried out about 30–40 years previously, except for a few studies related to GC-MS analysis. Detailed bioassay guided isolation studies may afford pharmacologically active compounds. Extracts of different parts of *C. hirsutus* were evaluated for their antimicrobial, antidiabetic, immunomodulatory, hepatoprotective activities among others. However, most of these studies were based on in vitro evaluation methods and only a few studies were carried out in animal models. There are also not many studies evaluating the activity of isolated compounds. In the future, properly designed in vivo and clinical studies are necessary to evaluate the pharmacological activities of *C. hirsutus* along with bioassay guided chemical isolation studies to isolate and identify the active constituents. Similarly, the safety and toxicity evaluation studies are not performed in detail. The possible herb–drug interaction should also be studied in detail in the future. 

## Figures and Tables

**Table 1:** List of reported alkaloids from form *C. hirsutus.*

S.N.	Compound Name	Plant Part/Extract	Reference
**1**	Jamtinine	Whole plant/ethanol extract	[5,22]
**2**	Jamtine *N*-oxide	Stems and roots	[22,42]
**3**	Haiderine	Whole plant/ethanol extract	[22]
**4**	Hirsutine	Whole plant/ethanol extract	[22,43]
**5**	Cohirsitine	Whole plant/ethanol extract	[22]
**6**	Cohirsitinine	Whole plant/ethanol extract	[22,44]
**7**	Cohirsine	Whole plant/ethanol extract	[22,45]
**8**	Cohirsinine	Whole plant/ethanol extract	[22,46]
**9**	Corsutine	Stems and roots/ethanol extract	[47]
**10**	Coclaurine	Stems and roots, whole plant/ethanol extract	[22,33,34]
**11**	Shaheenine	Stems and roots	[22,43]
**12**	Magnoflorine	Stems and roots	[22,33]
**13**	Trilobine	Stems and roots	[22,33,34]
**14**	Isotrilobine	Stems and roots	[22,33]
**15**	Cocsuline-*N*-2-oxide	Whole plant/ ethanol extract	[48]
